# Tobacco and COVID-19: Understanding the science and policy implications

**DOI:** 10.18332/tid/131035

**Published:** 2020-12-14

**Authors:** Luke Clancy, Silvano Gallus, Janice Leung, Catherine O. Egbe

**Affiliations:** 1 TobaccoFree Research Institute Ireland, Dublin, Ireland; 2 Department of Environmental Health Sciences, Istituto di Ricerche Farmacologiche Mario Negri IRCCS, Milan, Italy; 3 Centre for Heart Lung Innovation, The University of British Columbia and St. Paul’s Hospital, Vancouver, Canada; 4 Alcohol, Tobacco and Other Drug Research Unit, South African Medical Research Council, Pretoria, South Africa; 5 Department of Public Health, Sefako Makgatho Health Sciences University, Pretoria, South Africa

**Keywords:** COVID-19, smoking, tobacco control, ACE-2, tobacco sales ban

*The 18th WCTOH has announced its plan to hold a virtual Leadership Summit on Tobacco Control from 6–7 May 2021. Ahead of the Summit it is planned to hold a series of open-access webinars to provide on-going access to expert opinion on issues of importance to the Tobacco Control community. The first of these webinars entitled ‘Tobacco and COVID-19: Understanding the science and policy implications’, was held on 29th September 2020. During the webinar, Silvano Gallus, Janice Leung and Catherine Egbe were the invited expert speakers*.

There is an urgency to understand the relationship between COVID-19 and tobacco use. Smokers with diseases where tobacco is a major causative factor, e.g. COPD, ischaemic heart disease, cancers and diabetes were all quickly recognized as significant risks for poor prognosis in COVID-19. The effect of smoking itself may be more nuanced. There has not appeared to be increased prevalence of the disease, which was surprising as other respiratory viruses were well known to cause more disease in smokers. But published results seemed to show increased severity and increased mortality of COVID-19 in smokers. There is a large number of scientific publications on the topic in the preprint and peer-reviewed literature that is of varying quality and reliability. The relationship between SARS-CoV-2 and ACE-2 has also led to a number of studies exploring the possible mechanism of the relationship between smoking and ACE-2, but again it is not clear-cut. In the background is the worry that Tobacco Control like many other public health priorities would be side-lined during the pandemic despite tobacco causing some seen million deaths per annum. Not only that, but it seemed likely that the tobacco industry would take the opportunity to further its commercial aims amid the current uncertainty and distress and the neglect of Tobacco Control caused by the health emergency. The 18th WCOTH recently took the opportunity to explore these issues in an open-access webinar.

## Smoking, COVID-19 incidence and progression

There is strong evidence that smoking has a detrimental effect on the progression of COVID-19 and mortality^1-3^. A meta-analysis by Patanavanich and Glantz^2^ showed that, among COVID-19 patients, the risk of progression (severity or mortality) was significantly higher (OR=1.91; 95% CI: 1.42–2.59) for ever smokers compared with never smokers. As regards smoking prevalence among samples of COVID-19 patients, a number of case-series published in peer-reviewed journals or preprints showed a particularly low smoking prevalence compared to national estimates in the general adult population^3^. However, the large majority of these studies suffered from various forms of selection and information biases. Moreover, these studies by design do not have a comparison group and consequently cannot be used to support any causal conclusion. The evidence should rely on cohort studies analyzing the association between smoking and COVID-19 incidence. Simons et al.^3^ found at least 17 of those cohorts, providing a pooled relative risk (RR) of 0.74 (95% CI: 0.58–0.93) for current and 1.05 (95% CI: 0.95–1.17) for former smokers. However, there are limitations in several studies, including the relatively limited quality of most published studies, limited sample size and selection bias due to an over-representation of current smokers in study populations. More importantly, many of the cohorts were based on participants selected among those subjects voluntarily going to be tested for COVID-19, most likely because they had respiratory symptoms. Given that smokers are tested more frequently than never smokers^4^, these studies suffer from an important selection bias due to an over-representation of current smokers in their populations. Consequently, these cohorts show an apparent decreased risk of infection for current versus never smokers, even assuming no association between smoking and COVID-19 incidence. These limitations may totally or partially explain the apparent protective effect on the incidence of COVID-19 for current smokers. In view of the outcomes as regards severity and mortality, preventing COVID-19 complications appears to be an additional good reason to avoid smoking and to recommend smoking cessation.

## ACE-2 and the entry of SARS-CoV-2

Within human physiology there is a balance in the Renin/Angiotensin system in the body and the actions of Angiotensin Converting Enzyme (ACE) and of Angiotensin Converting Enzyme 2 (ACE-2). Current evidence indicates that the relationship between ACE-2 and the entry of SARS-CoV-2 into pulmonary tissue seemed to be mediated by TMTRSS2 activation through the Spike (S) protein on the virus. Previous research from the St. Paul’s Hospital Bronchoscopy Biobank has demonstrated the upregulation of ACE-2 that occurs in small airway epithelium (SAE), trachea and mucous cells in smokers and patients with COPD-19^5^. The results showed a dose-response relationship with smoking status such that ACE-2 expression was higher in current than former smokers than never smokers with the ACE-2 levels found to be related to the degree of pulmonary function impairment with a greater deficit showing higher ACE-2 levels. These findings are corroborated by those of other publications, including a meta-analysis from Cai et al.^6^ showing similar findings^7,8^. Notably, although further work is needed to define the role of nicotine or indeed upregulation of ACE-2^9^, it is possible that ACE-2 upregulation may explain the increased risk of severe COVID-19 in these populations, highlighting the importance of smoking cessation for these individuals and increased surveillance of these risk subgroups for prevention and rapid diagnosis of this potentially deadly disease.

## Opportunities and threats to tobacco control policies during COVID-19

COVID-19 poses a unique opportunity for combatting tobacco use. Notably, among policies implemented during the lockdown in countries such as South Africa, India and Botswana was a ban on the sale of tobacco and nicotine products^10^. Lockdown tobacco bans have had both positive and negative impacts on tobacco control. In South Africa for example, the lockdown tobacco ban caused many smokers to quit smoking or reduce the number of cigarettes smoked per day^11^. Even in countries where tobacco sales bans were not imposed, studies show that there has been a record number of smokers quitting as a result of the COVID-19 pandemic and the link between tobacco use and developing worse symptoms of COVID-19^12,13^. Also, there has been an increase in quit attempts and intention to quit. During this period, the World Health Organization also launched *Meet Florence,* the first artificial intelligence quit tobacco initiative. In general, one positive aspect of the pandemic is that more people have become aware of the harms of tobacco to health and wellbeing. On the contrary, it has been claimed that tobacco sales ban during COVID-19 lockdowns have led to a further expansion of the South Africa’s illicit tobacco market^11^. Furthermore, the COVID-19 pandemic has led to unprecedented loss of jobs and sharp decline in national economies globally. This also means that more people are facing financial and psychological stress during this time^14^. Research in some countries has shown that some smokers increased the number of cigarettes smoked per day during the pandemic^14^. A newspaper report also revealed that Bhutan, which is the only country in the world where cigarettes are illegal, decided to lift the ban on tobacco to prevent the spread of SARS-CoV-2 from neighboring India^15^. Around the world, the tobacco industry exploited the concerns around the pandemic to seek partnership with governments as well as inducing customers through gifts and promotional items^16,17^.

The lessons that we need to learn from the COVID-19 pandemic include the role of tobacco in the burden of non-communicable diseases and some infectious diseases. It also highlights the importance of Article 5.3 of the WHO Framework Convention on Tobacco Control (FCTC) and the need for measures to ensure its implementation at country level to address tobacco industry interference. The possible expansion of the illicit tobacco market during the pandemic highlights the importance of countries becoming a party to the protocol to eliminate illicit trade in tobacco products in order to put in place evidence-based measures to formally tackle illicit tobacco trade. Governments (especially in low- and middle-income countries) need to review their approach to smoking cessation. There is need for a coordinated response to the tobacco epidemic at country level, which should include improved access to smoking cessation aids and programs. The issue of finding alternative means of livelihood for tobacco farmers has also come to bear at this time on job losses. The tobacco industry has used their role as job creators to push for the lifting of tobacco bans during lockdowns. Article 17 of the WHO FCTC encourages countries to find alternative means of livelihood for tobacco farmers and this would be a good time to think about how to get this done in countries highly dependent on tobacco farming.

We have gleaned a lot of information in a short time but we certainly are only at the beginning of our understanding of SARS-CoV-2 and COVID-19 and tobacco and nicotine. Better planned and structured studies should now be implemented to more fully understand the role of smoking in COVID-19, with data on smoking and other tobacco products in COVID-19 collected and analyzed with attention to avoiding the many biases that present data reveal.

However, there is also the best opportunity in two centuries to stop the devastation caused by tobacco^18^. The whole world has shown the importance they attach to health and life. The acceptance of the loss of personal freedoms and the huge financial price that society is willing to pay to try to prevent the pandemic is a clear signal to deal with tobacco. The market value of the tobacco industry worldwide is said to be some US$1 trillion by the year 2026^19^ whereas COVID-19 has cost some $9 trillion dollars in the Group of 20 nations by May this year alone, and is still rising^20^. According to the International Labour Organization (ILO), the COVID-19 crisis will wipe out 6.7% of working hours globally in the second quarter of 2020, equivalent to 195 million full-time workers^21^ compared with a million workers directly or indirectly employed in the tobacco industry. Now must be the time to declare that we will not tolerate 7 million deaths per year every year from tobacco addiction, an entirely preventable disease which is due, not to a virus like SARS-CoV-2 which may cause 1.5 million COVID-19 deaths this year^22^, but to the deliberate actions of a greedy industry owned by a small number of international companies. We need to transform and eventually close down this industry^23^. If the 20th century was the Cigarette Century^24^ the 21st should be the Tobacco-Free Century.
